# ﻿An overlooked morphological feature in the genus *Rhodospatha* (Araceae, Monsteroideae, Anepsiadeae) revealed through international collaboration, with the description of a new endemic species from Costa Rica

**DOI:** 10.3897/phytokeys.260.154762

**Published:** 2025-07-28

**Authors:** Marco Cedeño-Fonseca, Oscar Cubero-Vásquez, Orlando O. Ortiz, Marilyn Rodríguez-Arias, Maria Alejandra Serna-Sánchez, Edwin Trujillo-Trujillo, José Esteban Jiménez, Alejandro Zuluaga, Alistair Hay

**Affiliations:** 1 Botanischer Garten und Botanisches Museum Berlin, Freie Universität Berlin, Königin-Luise-Straße 6-8, D-14195 Berlin, Germany Freie Universität Berlin Berlin Germany; 2 Centro de Investigación Jardín Botánico Lankester, Universidad de Costa Rica, P.O. Box 302-7050, Cartago, Costa Rica Universidad de Costa Rica Cartago Costa Rica; 3 Research Associate, Herbario Luis Fournier Origgi (USJ), Centro de Investigación en Biodiversidad y Ecología Tropical, Universidad de Costa Rica, Apdo.11501−2060, San José, Costa Rica Universidad de Costa Rica San José Costa Rica; 4 Universidad Nacional, Instituto Internacional en Conservación y Manejo de Vida Silvestre (ICOMVIS), Maestría en Manejo y conservación de Vida Silvestre, Heredia, Costa Rica Cloudbrige Nature Reserve, San Gerardo de Rivas San Jose Costa Rica; 5 Cloudbrige Nature Reserve, San Gerardo de Rivas, Pérez Zeledón, Apdo. 11904, San Jose, Costa Rica Universidad Nacional Heredia Costa Rica; 6 Universidad de Panamá, Herbario PMA, Estafeta Universitaria, Apdo. 3366, Panama City, Panama Universidad de Panamá Panama City Panama; 7 Coiba Scientific Station (COIBA AIP), Clayton, Ciudad del Saber, Panama Coiba Scientific Station (COIBA AIP) Clayton, Ciudad del Saber Panama; 8 Grupo de Investigación Schultes, Fundación Ecotonos, Valle del Cauca, Cali, Colombia Grupo de Investigación Schultes, Fundación Ecotonos Cali Colombia; 9 Department of Biology, University of Missouri-St. Louis, St. Louis, MO, USA University of Missouri- St. Louis St. Louis United States of America; 10 Laboratorio de Agrobiodiversidad y Malherbologia LAMUA. Doctorado en Ciencias Naturales y Desarrollo Sustentable, Universidad de la Amazonia, Florencia, Colombia Universidad de la Amazonia Florencia Colombia; 11 Florida Museum of Natural History, University of Florida Herbarium, 379 Dickinson Hall, 1659 Museum Rd., Gainesville, Florida 32611, USA University of Florida Herbarium Gainesville United States of America; 12 Department of Biology, University of Florida, Gainesville, Florida 32611, USA University of Florida Gainesville United States of America; 13 Departamento de Biología, Herbario de la Universidad del Valle, Universidad del Valle, Calle 13 # 100-00, Cali, Colombia Universidad del Valle Cali Colombia; 14 Royal Botanic Gardens Sydney, Mrs Macquarie's Road, Sydney 2000, Australia Royal Botanic Gardens Sydney Sydney Australia; 15 Jardín Botánico de la Paz y Flora, Bitaco, Valle del Cauca, Colombia Jardín Botánico de la Paz y Flora Bitaco Colombia

**Keywords:** Cloudbridge Nature Reserve, Monsteroideae, Pérez Zeledón, Talamanca, taxonomy

## Abstract

A recent floristic survey in the Pacific slope of the Talamanca Mountain (Costa Rica), has revealed a new species of *Rhodospatha*, characterised by a bracteolate inflorescence. We describe and fully illustrate *Rhodospatharubrinervis* from the Cloudbridge Nature Reserve. A detailed taxonomic description, as well as its distribution, ecology, phenology and conservation assessment are provided, along with a comparative discussion of its morphological affinities with *R.forgetii* and *R.wendlandii*. In addition, we present a brief discussion on the presence of a bracteole in *Rhodospatha*, a morphological character that, although illustrated by H.W. Schott 160 years ago, has not previously been described in detail.

## ﻿Introduction

The Neotropics are the most species-rich region on Earth, harbouring approximately 37% of the world’s plant species (Antonelli and Sanmartín 2011). Climbing plants represent an important key component of this biodiversity and are highly diverse in tropical forests (Gentry 1991; Sperotto et al. 2020). Amongst the ten most diverse families of climbing plants is the family Araceae Juss. (Gentry 1991), which also ranks as one of the largest monocot families (Croat 2019). Within Araceae, the neotropical genus *Rhodospatha* Poepp. displays a substantial diversity.

*Rhodospatha* belongs to the subfamily Monsteroideae Schott (Mayo et al. 1997) tribe Anepsiadeae Schott (Zuluaga et al. 2019; Haigh et al. 2023). *Rhodospatha* is typical of tropical wet forests, comprising predominantly appressed-climbing nomadic vines, although some species are terrestrial or rheophytic (Mayo et al. 1997; Grayum 2003; Alzate-Lozano et al. 2019). While primarily a lowland genus, mostly distributed in the Amazon Basin, *Rhodospatha* can reach elevations of up to 2200 m a.s.l. in Central America (Grayum 2003; Croat and Delannay 2024; Delannay and Croat 2024) and Colombia. The genus ranges from southern Mexico to Bolivia and the Caribbean islands of Trinidad and Tobago (Mayo et al. 1997; Grayum 2003; Delannay and Croat 2024). Diagnostic features of the genus include entire, mostly membranous leaf blades, a frequently pink to red spathe, axile placentation of the ovary with (2)3-many ovules per locule and lenticular, strongly curved and flattened seeds, less than 3 mm long with endosperm present (Mayo et al. 1997; Croat and Delannay 2024; Delannay and Croat 2024).

The known diversity of *Rhodospatha* has increased significantly in recent years due to the description of many new species (Temponi et al. 2012; Alzate-Lozano et al. 2019; Cedeño-Fonseca et al. 2023; Croat and Delannay 2024; Delannay and Croat 2024). Boyce and Croat (2023) suggested 28 accepted species with 70 more anticipated, while Delannay and Croat (2024) proposed a total of 136 species in their monographic treatment of the genus. In Central America, *Rhodospatha* is represented by 19 species, with only *R.wendlandii* Schott extending as far north as southern Mexico (Croat and Delannay 2024; Delannay and Croat 2024). Approximately half of this diversity occurs in Costa Rica (Croat and Delannay 2024). Grayum (2003) recognised nine species in Costa Rica, five of which were formally described only in recent years by Cedeño-Fonseca et al. (2023) and Croat and Delannay (2024). Currently, Croat and Delannay (2024) list ten species of *Rhodospatha* occurring in the country.

Despite recent advances in species descriptions by Cedeño-Fonseca et al. (2023), the taxonomic revision by Croat and Delannay (2024) and the monographic work by Delannay and Croat (2024), descriptions of inflorescence morphology within the subfamily Monsteroideae have consistently followed a generalised pattern: a single peduncle, an erect, often coloured spathe, a spadix bearing bisexual flowers and the presence or absence of the sterile zone either at the base or apex of the spadix (Mayo et al. 1997: 125). However, recent international collaborative work, involving the examination of hundreds of herbarium specimens, historical collections and recent fieldwork in Costa Rica, Panama and Colombia, has revealed the presence of an overlooked structure at the apex of the peduncle in *Rhodospatha*, which has not previously been reported or described for the Araceae. Therefore, in this article, we provide a complete description of a new *Rhodospatha* species endemic to Costa Rica with a bracteolate inflorescence, document the presence of spathe-subtending bracts in this genus and list the species in which such structures have been observed by us.

## ﻿Material and methods

The fieldwork was carried out between April 2023 and October 2024 in the Cloudbridge Nature Reserve (Talamanca Mountains of Costa Rica). Specimens were collected in the field using tall pruners and hand pruners and prepared as herbarium vouchers. Location and elevation data were recorded using a Garmin GPSMAP64sc. Morphological assessments of the living plant were based on the comparison of juvenile, pre-adult and adult plants, as well as documentation of inflorescences both in development and at anthesis. The collected samples were processed and deposited at the Dr. Luis A. Fournier Origgi (USJ) Herbarium, University of Costa Rica.

Morphological measurements were taken from both living plants and herbarium specimens. Photographs of living plants were captured with a Canon PowerShot SX540HS digital camera, as well as with a high-resolution iPhone 11 Pro phone camera. The plates were prepared using the Lankester Composite Dissection Plate (LCDP) methodology (Karremans et al. 2020) and edited with Adobe Photoshop (versions 2021–2023). Additionally, all relevant taxonomic literature from Mesoamerica and South America, as well as type specimens available through JSTOR Global Plants (2022) and protologues, were examined.

The following Herbaria (acronyms follow Thiers (2025) continuously updated) were consulted: AGUAT, B, BM, BIGU, CHIP, COAH, COL, CR, CSAT, CUVC, DUKE, ENCB, F, HEM, HLDG, HNMN, HUAZ, ITIC, JAUM, JBB, JVR, K, LAGU, LSCR, MA, MEXU, MHES, MO, NY, PMA, SEL, SCZ, TEFH, UDBC, UCH, UJUAT, USCG, USJ, XAL and WU, as well as online specimen images available from C, COL, EAP, ENCB, MEXU and TEFH.

For the documentation of the number of locules and ovules, an inflorescence from each species was selected. Subsequently, four flowers per inflorescence were dissected under a Leica stereoscope (LEICA-DUAL-MZ6), using titanium tweezers to ensure precision. Each flower was longitudinally incised from apex to base to expose the locules. After identification, each locule was extracted using tweezers and 70% alcohol was added to improve visibility and enable accurate ovule counting. To mitigate rapid evaporation of the alcohol, periodic application of water was used to maintain optimal visibility of the structures. This procedure was performed in the laboratories of the Lankester Botanical Garden.

The conservation status was evaluated, based on IUCN Red List Categories and Criteria (IUCN 2012) and following the guidelines of the IUCN Standards and Petitions Committee (2022). The Extension of Occurrence (EOO) was estimated across GeoCAT (Bachman et al. 2011). Life zones are according to the terminology by Holdridge et al. (1971).

## ﻿Taxonomic treatment

### ﻿ Rhodospatha rubrinervis

Taxon classificationPlantaeAraceaeMonsteroideae

M.Cedeño, O.Cubero & O.Ortiz sp. nov.

EA9857B1-5EF0-598E-92CA-8378040EB4BB

urn:lsid:ipni.org:names:77366293-1

Figs 1 , 2 , 3

#### Type.

Costa Rica • San José: Cantón Pérez Zeledón, distrito de Rivas, San Gerardo, Cloudbrige Nature Reserve, 1800 m elev., 26 Apr 2023, *O. Cubero, M. Rodríguez-Arias & M. Cedeño 005* (holotype: USJ!; isotype: CR!).

**Figure 1.**
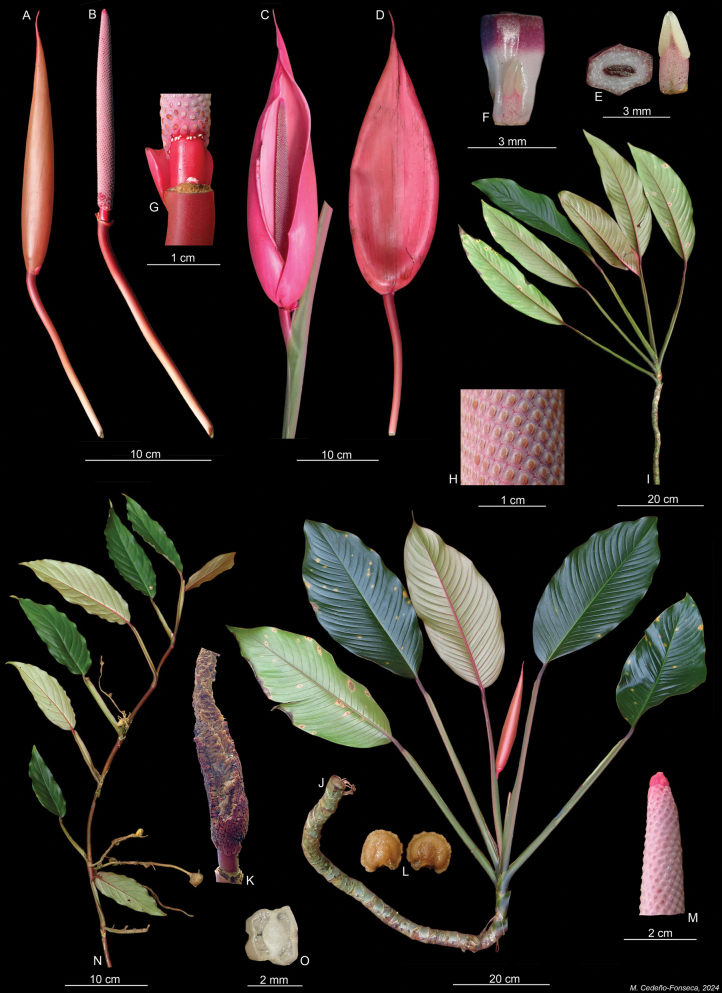
*Rhodospatharubrinervis*. **A.** Developing inflorescence; **B.** Spadix in female anthesis; **C.** Inflorescence with spathe, in female anthesis; **D.** Inflorescence with open spathe (back view); **E.** Stylar plate with stigma (left) and one stamen (right); **F.** Fertile flower, in lateral view; **G.** Bracteole at the apex of the peduncle; **H.** Portion of spadix with stigmas in female anthesis; **I.** Pre-adult plant; **J.** Portion of adult plant; **K.** Mature infructescence; **L.** Seeds; **M.** Spadix with pink sterile region at the apex; **N.** Juvenile plant; **O.** Transversal section of the ovary with two locules. Photos **A–D, I, J, K, L** by O. Cubero; **E–H, K–O M** by M. Cedeño. *O. Cubero 005* (USJ).

**Diagnosis**. *Rhodospatharubrinervis* is similar to *Rhodospathaforgetii* N.E.Br. (Fig. 4A–M), but differs in having internodes 1–7 cm long, salmon-coloured dark green or light green (vs. 6–8 cm long, dark green), petiole green at the base with a conspicuous longitudinal bright pink band or line abaxially towards the geniculum (vs. petiole light green without band or line abaxially, Fig. 5B), geniculum bright pink or dark purple, sometimes with green dots (vs. geniculum grooved always green, Fig. 5D), leaf blades light green with lilac and white punctations on the lower surfaces (vs. light green with white punctations, Fig. 5E), mid-rib bright pink up to the apex (vs. constantly green, Fig. 5C), primary lateral veins bright pink up to the middle and green from the middle to margin (vs. green along the whole length, Fig. 5C), spathe-subtending bracteole reddish with constriction line near the margin (vs. pinkish without constriction line, Fig. 6A–F); spathe brownish-bright pink externally during development, bright pink externally and internally at anthesis (vs. brownish-orange externally during development, Fig. 4A, pale pink externally and internally at anthesis, Fig. 4B, J, N), spathe up to 15.3 cm longer than the spadix and deciduous (vs. up to 6 cm longer than the spadix and marcescent, Fig. 5A), stipe red to bright pink, up to 1.7 cm (vs. brown, sessile or stipitate up to 6 mm, Fig. 4C), spadices pinkish during development and anthesis (vs. lilac during development and pinkish at anthesis, Fig. 4C, D, F), stamens with pinkish laminar filament (vs. creamy-white, Fig. 4H) and ovary with up to 30 ovules per locule (vs. up to 40 ovules, Fig. 5F).

**Figure 2.**
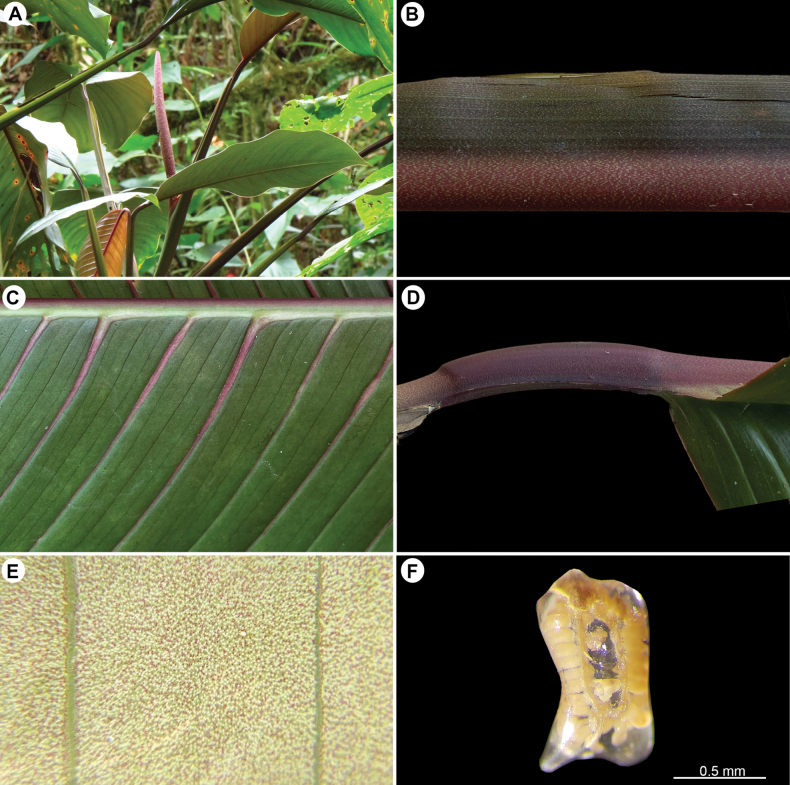
*Rhodospatharubrinervis*. **A.** Adult plant with inflorescence in male anthesis without spathe; **B.** Portion of the petiole with green punctuations and persistent petiolar sheath; **C.** Mid-rib convex abaxially and primary lateral veins with bright pink colour up to the middle; **D.** Geniculum dark purple; **E.** Abaxial surface light green with lilac and white punctuations; **F.** Longitudinal section of ovary showing 30 ovules per locule along axile placentas. Photos **A** by O. Cubero; **B–E** by M. Cedeño; **F** by A. Serna.

**Figure 3.**
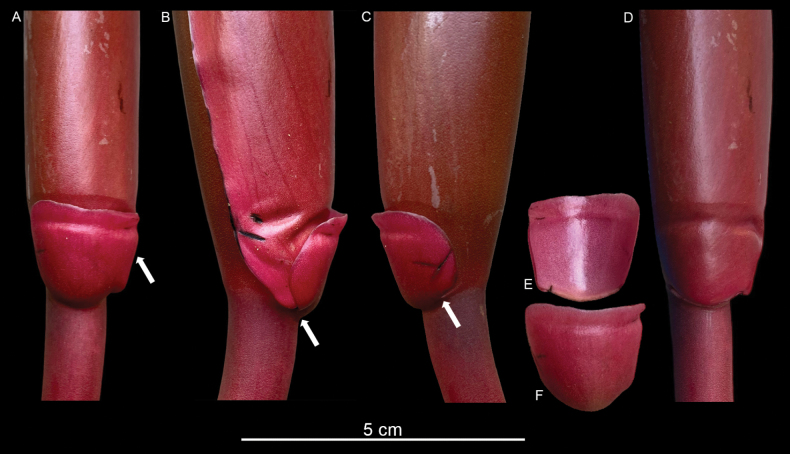
Bracteolate inflorescence in *Rhodospatharubrinervis*. **A.** Frontal view of bracteole at the base of the spadix, with constriction line near margin (white arrow); **B.** Left view of the bracteole free of the spathe (white arrow); **C.** Right view of the bracteole free of the spathe (white arrow); **D.** Inflorescence showing scar of the bracteole; **E.** Back view of the bracteole; **F.** Frontal view of the bracteole. Photos by M. Cedeño.

**Figure 4.**
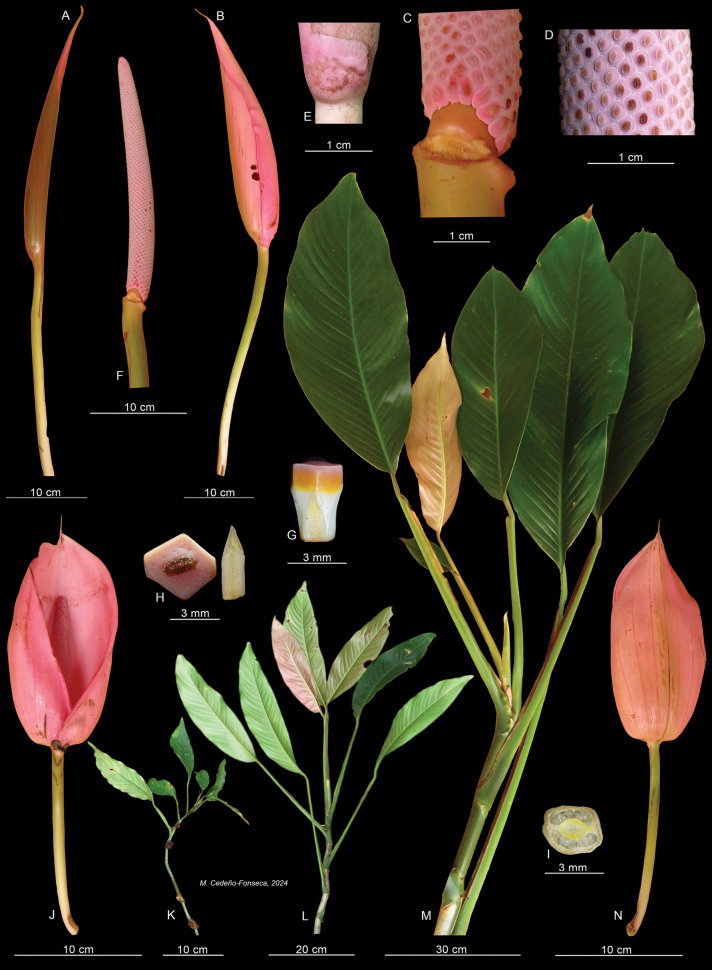
*Rhodospathaforgetii*. **A.** Developing inflorescence; **B.** Inflorescence with pink spathe in female anthesis; **C.** Spadix with stipe and flowers in female anthesis; **D.** Portion of the spadix with stigmas in female anthesis; **E.** Bracteole at the apex of peduncle; **F.** Spadix in female anthesis; **G.** Fertile flower in lateral view; **H.** Stylar plate with stigma (left) and one stamen (right); **I.** Transverse section of the ovary with two locules; **J.** Inflorescence with open spathe front view; **K.** Juvenile plant; **L.** Pre-adult plant; **M.** Portion of the adult plant; **N.** Inflorescence with open spathe back view. Photos **A–C, F, J–N** by O. Cubero; **D, E, G–I** by M. Cedeño. *O. Cubero 001* (USJ). Modified plate from Cedeño-Fonseca et al. (2023).

**Figure 5.**
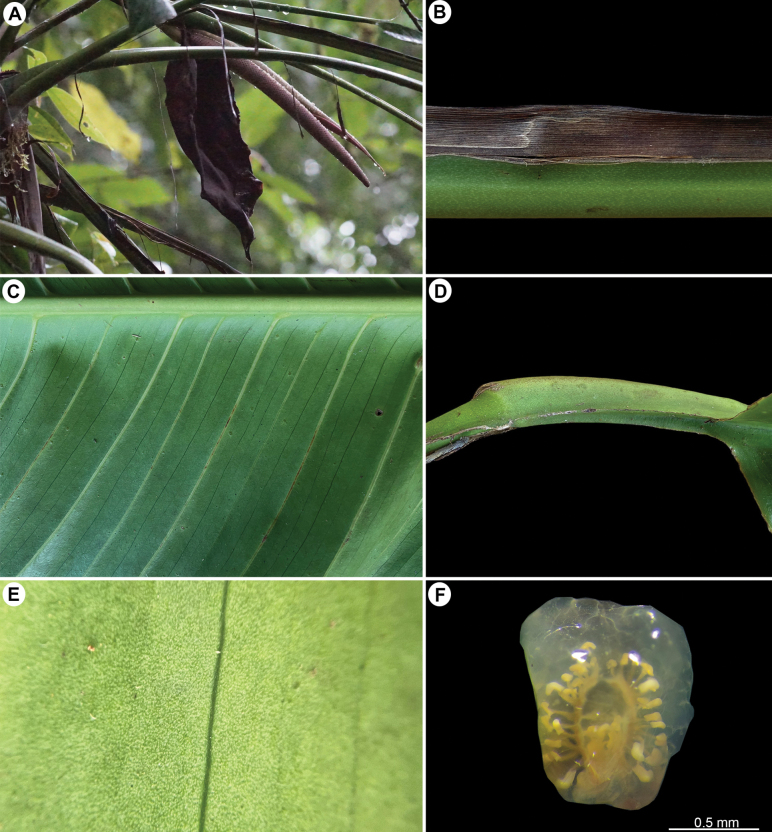
*Rhodospathaforgetii*. **A.** Adult plant with inflorescence in male anthesis with marcescent spathe; **B.** Portion of the petiole with green colour and marcescens petiolar sheath (proximally to be deciduous); **C.** Abaxial mid-rib convex, primary lateral veins green to the margin; **D.** Geniculum green; **E.** Abaxial surface light green with white punctuations; **F.** Ovary in longitudinal section with 40 ovules per locule along axile placentas. Photos **A–E** by M. Cedeño; **F** by A. Serna.

#### Description.

Robust nomadic vine, appressed-climbing. ***Seedlings***: unknown. ***Juvenile plants***: terrestrial appressed; ***stem*** cylindrical, shiny and smooth, dark green or dark purple, with light green dots; ***internodes*** 3–7 cm long and 7–9 cm diam.; ***petiole*** smooth, 7–30 cm long, light green changing to bright pink at the base and near to the geniculum; ***petiole sheath*** persistent; ***geniculum*** bright pink with green dots; ***leaf blades*** 12–25 × 4.5–8.0 cm, lanceolate to elliptical, non-equilateral, acute to narrowly rounded at the base and long acuminate at the apex, reddish to pinkish when new, changing to shiny dark green in the upper surface and light green in lower surface, decurrent on geniculum; ***mid-rib*** sunken adaxially, convex abaxially and bright pink; primary lateral veins bright pink; ***margin*** with a reddish line on the edge. ADULT PLANTS: root climbers and branching, growing up to 7 m above ground; ***stem*** cylindrical or slightly flattened, shiny, smooth, dark green, light green sometimes and becoming salmon-coloured in some areas; ***internodes*** 1–7 cm long, 7–9 cm in diam.; ***anchor roots*** light green and sometimes green mixed with purple; ***feeder roots*** light green and sometimes green mixed with violet; ***petiole*** 24–71 cm long, smooth, dark green at the base and mostly becoming light green, with a conspicuous longitudinal bright pink band or line abaxially up to geniculum, sometimes with small salmon-coloured areas mainly in new leaves; sheathed to base of geniculum; ***petiole sheath*** persistent or sometimes becoming deciduous with fibrous remnants; ***geniculum*** 2–6 cm long, dark purple sometimes with green punctations, sunken adaxially, convex abaxially; ***leaf blades*** 33–82 × 12–30 cm, oblong or lanceolate to elliptical, non-equilateral, subcoriaceous, cuneate to attenuate at the base, acuminate at the apex, shiny dark green above and light green below with lilac and white punctations, decurrent at geniculum, drying blackish- or greenish-yellow with reddish-bright pink primary lateral veins; ***mid-rib*** bright pink, sunken adaxially, convex abaxially; ***primary lateral veins*** 24–29 per side, bright pink up to the middle and green from the middle to margin, sunken adaxially, prominent abaxially; ***interprimary veins*** prominent and parallel towards margin, in young blade primary and interprimary veins intense bright pink; ***collective vein*** not visible; ***margin*** undulate and sometimes reddish. INFLORESCENCES on ascending stems, several simultaneously in flowering season, arranged in leaf axils or within a green cataphyll; ***peduncle*** smooth, reddish, 19–25.5 cm long, 2–4 mm diam.; ***bracteole*** subtending spathe (not peduncle) 1.0–1.5 × 1.7–2.0 cm, reddish with constriction line near to margin; ***spathe*** acuminate to acuminate, 28–38.5 cm long, 6–9 cm wide, brownish-bright pink externally during development, bright pink externally and internally at anthesis, up to 15.3 cm longer than spadix, membranous, completely open, with overlapping margins at base, deciduous a few hours after opening; ***stipe*** bright red to bright pink up to 1.1–1.7 cm.; ***spadix*** 18.8–23.2 cm long, 4.4–5.3 cm in diam., cylindrical and weakly tapered to apex, pinkish during development and anthesis, with red sterile region at apex, with 19–21 flowers in principal spiral and 11–12 in secondary spiral, ***flowers*** 5–6 mm long; stamens 1.5 to 5.0 mm long, with pinkish laminar filaments; anthers 0.5 to 1.0 mm long; ovary quadrangular in longitudinal section, 2–3 × 1.3–2.0 mm, bilocular with up to 30 ovules per locule, borne on axillary placentas; style quadrangular or hexagonal, 2.0–2.5 × 1.8–2.0 mm; stigma linear with transparent stigmatic secretion; berries white and pinkish, seeds light brown, reniform.

#### Etymology.

The species epithet “*rubrinervis*” refers to the distinctive reddish colouration of the mid-rib and primary lateral veins. This striking venation not only serves as a key diagnostic character in the field, but also highlights the aesthetic appeal and uniqueness of the species within the genus *Rhodospatha*.

#### Distribution and ecology.

This species is endemic to Costa Rica, occurring in the region of Pérez Zeledón on the Pacific slope of the Cordillera de Talamanca, and in Bosque Tropical Nuboso Palo Verde on the Caribbean slope. It grows in cloud forests and premontane rain forest life zones, in mature secondary forest, at around 1800 m elevation.

#### Phenology.

Flowering time from March to June; fruits have been recorded in September and October.

#### Conservation status.

The recent collections of *Rhodospatharubrinervis* reported here come from two localities within a protected area (Cloudbridge Nature Reserve and Bosque Tropical Nuboso Palo Verde, Costa Rica). Further information on the species’ distribution and population status is needed to enable more accurate conservation assessment. *Rhodospatharubrinervis* is, therefore, currently considered as Data Deficient (DD).

#### Discussion.

*Rhodospatharubrinervis* is characterised as follows: juvenile plants with internodes dark purple with light green dots; petiole sheath persistent, light green and bright pink at base; adult plants with internodes bright green, sometimes displaying small salmon-coloured areas; petiole sheath persistent; geniculum dark purple or bright pink; mid-rib bright pink; primary lateral veins bright pink up to the middle, becoming more intense in new emerging leaves; peduncles and bracteoles bright pink; spathes brownish-bright pink during development and bright pink both externally and internally at anthesis, up to 15.3 cm longer than spadix; stipe bright pink; spadix lilac with strongly pigmented pink sterile region at apex; 19–21 flowers arranged in main spiral and 11–12 in alternate spiral; stamens with pinkish laminar filaments; berries white to pinkish, seeds and reniform, light brown.

In addition to the differences cited in the diagnosis, another important feature distinguishing *Rhodospatharubrinervis* and *R.forgetii* lies in their growth pattern: *R.rubrinervis* has a greater abundance of leaves and shows the ability to branch and spread more widely on the host tree than *R.forgetii*, which rarely branches. Additionally, juveniles of *R.rubrinervis* are more frequently found on the forest floor than those of *R.forgetii*.

*Rhodospatharubrinervis* could be also confused with *Rhodospathawendlandii* Schott, but differs in having oblong or lanceolate to elliptical blades with 24–29 primary lateral veins that are bright pink up to the mid-point (vs. oblong-oblanceolate blades with 28–52 primary lateral veins, all green), bright pink mid-rib up to the apex (vs. entirely green mid-rib), reddish bracteole with a constriction line near the margin (vs. cream or white bracteole without constriction line); brownish-bright pink spathes externally during development, bright pink externally and internally at anthesis (vs. white to creamy throughout development and anthesis) and bright red to bright pink stipes (vs. white or cream).

Juveniles of *Rhodospatharubrinervis* may also be confused with those of *R.bogneri* Croat; however, *R.rubrinervis* can be distinguished by having a membranaceous leaf blade (vs. coriaceous), with undulate, reddish margins (vs. entire margins), a long-acuminate apex (vs. short-acuminate), and the base acute to narrowly rounded (vs. rounded).

#### Additional specimens examined.

**Costa Rica** • **San José**: cantón Pérez Zeledón, distrito de Rivas, San Gerardo, Reserva Biológica Cloudbridge, 1800 m elev., 26 Apr 2023, O. Cubero, M. Rodríguez-Arias & M. Cedeño 006 (USJ!); • San José: cantón Pérez Zeledón, distrito de Rivas, San Gerardo, Reserva Biológica Cloudbridge, 1800 m elev., 26 Apr 2023, O. Cubero, M. Rodríguez-Arias & M. Cedeño 007 (USJ!); • San José: cantón Pérez Zeledón, distrito de Rivas, San Gerardo, Reserva Biológica Cloudbridge, 1800 m elev., 26 Apr 2023, O. Cubero, M. Rodríguez-Arias & M. Cedeño 008 (USJ!), Cartago: cantón de Paraíso, distrito de Orosí, Navarro, 1410 m elev., 12 Oct 2024, O. Cubero, M. Rodríguez-Arias & M. Cedeño 032 (USJ!).

#### Remarks on the spathe-subtending bracteoles in *Rhodospatha*.

The morphology of leaf types in Araceae is diverse, including several forms of cataphylls and reduced leaves, which have been described in detail by Ray (1987a, 1987b). These modified leaves are usually found subtending the peduncles, particularly in taxa forming synflorescences (Ray 1987a, 1987b). However, we observed that, in several *Rhodospatha* species, there is a distinct bract-like structure directly subtending the spathe at the top of the peduncle. This peduncular bracteole seems to have been largely overlooked, although it was illustrated long ago by Schott, for example, in his painting of *Rhodospathawendlandii* [Schott 1864: iconography 2994, deposited in the Natural History Museum of Vienna, also available in the microfiche edition (Nicolson 1984); cited without remark in Croat and Delannay (2024): fig. 88] and mentioned in the protologue of that species (Schott 1864: 52) as the spathe base being ‘auriculate’. Engler and Krause (1908: 91–96) briefly alluded to short, persistent, bluntly triangular wings at the spathe base, again in reference to the same species. In line with these morphological observations, Madison (1978) noted that, in certain species from the northern Andes, the spathe bear two orbicular “flaps” at the base. Nonetheless, he did not provide a detailed characterization or formal definition of this structure. However, this structure has never been documented as being as widespread (though not universal) in the genus as we have found it to be. Its exact nature remains intriguing and we cannot yet provide a definitive explanation. Could it be interpreted as part of the spathe itself and, if so, what is its function or structural significance? Might it suggest peduncle elongation during development below, rather than above, a small subtending bracteole, somewhat analogous to the internodal elongation above or below the prophylls in the sympodial shoots of PhilodendronsubgenusPhilodendron vs. PhilodendronsubgenusMeconostigma (see Mayo 1991)?

**Figure 6.**
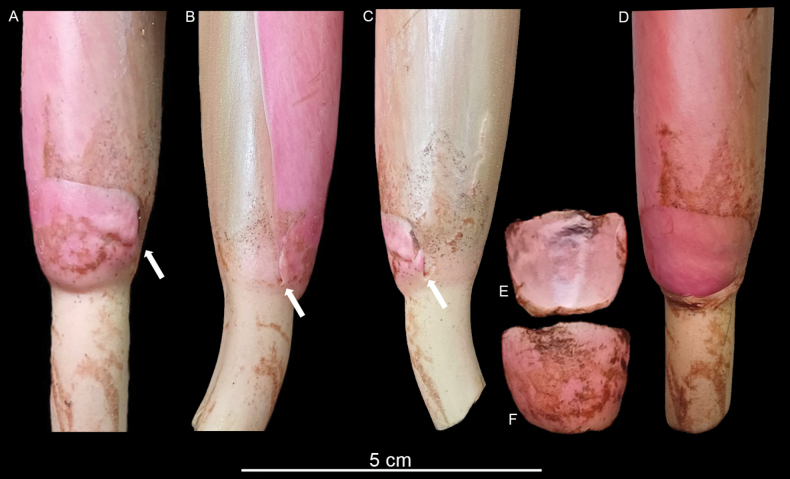
Bracteolate inflorescence in *Rhodospathaforgetii*. **A.** Front view of the bracteole in the base of the spadix (white arrow); **B.** Left view of the bracteole free of the spathe (white arrow); **C.** Right view of the bracteole free of the spathe (white arrow); **D.** Inflorescence with the scar of bracteole; **E.** Back view of the bracteole; **F.** Frontal view of the bracteole. Photos by O. Cubero.

**Figure 7.**
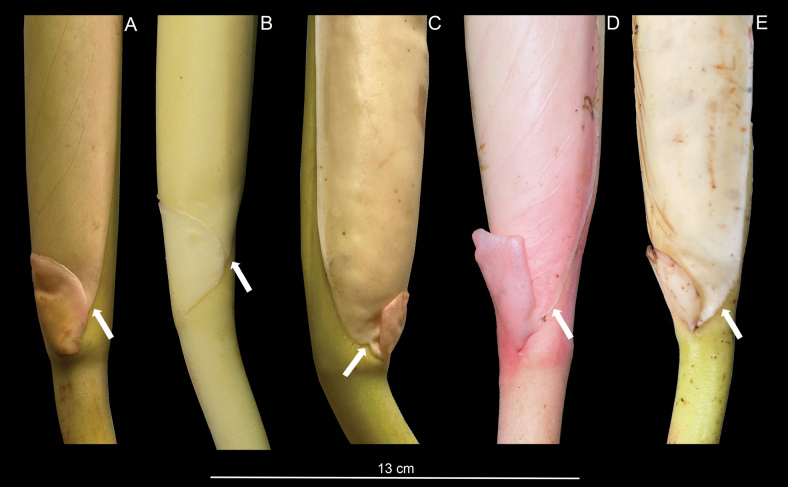
Bracteolate inflorescence in *Rhodospathawendlandii*. **A–E.** Inflorescences showing bracteolate structure at the base, in the same position when the spathe begins to open (white arrow). **A, B.** Inflorescence in development; **C**–**E.** Inflorescence in female anthesis. Photos by M. Cedeño.

We examined the inflorescence of *Rhodospathawendlandii* and found that this bract-like structure has the same position (Fig. 7A–E) as we observed in *R.rubrinervis* (Fig. 3B) and *R.forgetii* (Fig. 6B), always situated where the spathe begins to open and form the floral chamber. In *Rhodospatha*, a genus characterised by very early-deciduous and often fragile spathes, we observed that this bracteole seems to support the spathe during female anthesis, allowing the chamber to form without the base rupturing. The inflorescences of *R.wendlandii* and *R.rubrinervis* are visited by beetles of the genus *Cyclocephala* (Scarabaeidae) and these robust beetles can damage the spathe when entering the chamber. However, we detected that, when the beetles are inside, moving along the spadix, they can displace the spathe without causing it to open or break at the base, likely due to the presence of this bracteole. If the spathe were to break at the base, anthesis would end prematurely, as the spathe would rot, covering the spadix and preventing male anthesis from occurring. We hypothesise that a key structural function of this bracteole could be to act as a brake, preventing the spathe from tearing at the base when beetles enter in the female anthesis.

We have observed these structures in the following *Rhodospatha* species: *R.antonensis* Croat & O.Ortiz (Cedeño-Fonseca et al. 2023; fig. 1: voucher O.O. Ortiz 4319 PMA), *Rhodospathaforgetii* (voucher O. Cubero 001 USJ), *R.rubrinervis* (voucher: O. Cubero 005 USJ), *R.wendlandii* (voucher: O. Cubero 010 USJ) and *Rhodospatha* sp. (voucher: E. Trujillo 8410 HUAZ).

## Supplementary Material

XML Treatment for Rhodospatha rubrinervis

## ﻿Additional information

### Use of AI

No use of AI was reported.

### Author ORCIDs

Marco Cedeño-Fonseca https://orcid.org/0000-0003-0119-0427

Oscar Cubero Vásquez https://orcid.org/0009-0003-3626-8156

Orlando O. Ortiz https://orcid.org/0000-0002-7805-0046

Marilyn Rodríguez-Arias https://orcid.org/0009-0008-7944-7847

Maria Alejandra Serna-Sánchez https://orcid.org/0000-0002-7114-0591

Edwin Trujillo-Trujillo https://orcid.org/0000-0002-5601-540X

José Esteban Jiménez https://orcid.org/0000-0002-8154-2156

Alejandro Zuluaga https://orcid.org/0000-0002-5874-6353

Alistair Hay https://orcid.org/0000-0003-2884-6430

### Data availability

All of the data that support the findings of this study are available in the main text.
